# Consuming fresh macroalgae induces specific catabolic pathways, stress reactions and Type IX secretion in marine flavobacterial pioneer degraders

**DOI:** 10.1038/s41396-022-01251-6

**Published:** 2022-05-19

**Authors:** Maéva Brunet, Nolwen Le Duff, Tristan Barbeyron, François Thomas

**Affiliations:** Sorbonne Université, CNRS, Integrative Biology of Marine Models (LBI2M), Station Biologique de Roscoff (SBR), 29680 Roscoff, France

**Keywords:** Environmental microbiology, Marine microbiology, Microbial ecology

## Abstract

Macroalgae represent huge amounts of biomass worldwide, largely recycled by marine heterotrophic bacteria. We investigated the strategies of bacteria within the flavobacterial genus *Zobellia* to initiate the degradation of whole algal tissues, which has received little attention compared to the degradation of isolated polysaccharides. *Zobellia galactanivorans* Dsij^T^ has the capacity to use fresh brown macroalgae as a sole carbon source and extensively degrades algal tissues *via* the secretion of extracellular enzymes, even in the absence of physical contact with the algae. Co-cultures experiments with the non-degrading strain *Tenacibaculum aestuarii* SMK-4^T^ showed that *Z. galactanivorans* can act as a pioneer that initiates algal breakdown and shares public goods with other bacteria. A comparison of eight *Zobellia* strains, and strong transcriptomic shifts in *Z. galactanivorans* cells using fresh macroalgae vs. isolated polysaccharides, revealed potential overlooked traits of pioneer bacteria. Besides brown algal polysaccharide degradation, they notably include oxidative stress resistance proteins, type IX secretion system proteins and novel uncharacterized polysaccharide utilization loci. Overall, this work highlights the relevance of studying fresh macroalga degradation to fully understand the metabolic and ecological strategies of pioneer microbial degraders, key players in macroalgal biomass remineralization.

## Introduction

Macroalgae are major primary producers in coastal zones, acting as a global carbon sink [1]. Specific polysaccharides dominate macroalgal extracellular matrices (ECM) and can represent up to 50% of the dry weight [2]. For example, brown algae produce alginates and fucose-containing sulfated polysaccharides (FCSPs). Alginates are linear polymers of β-D-mannuronic (M) and α-L-guluronic acids (G), representing between 10 and 45% of the algal dry weight [2]. FCSPs, accounting for 4–13% of the dry weight [3], refer to linear or highly branched polysaccharides containing α-linked L-fucose residues together with a variety of other neutral monosaccharides constituents (e.g. galactose, mannose, xylose, rhamnose) and uronic acids [4]. They hold many substitutions, mainly sulfate and acetyl groups. The structure of brown algal polysaccharides is consequently highly heterogeneous and varies according to species, seasons, geographical locations, thallus part, algal growth stages and environmental factors [3–7]. Within the ECM, these carbohydrates are cross-linked and associated with proteins (3–15%), minerals (7–36% such as iodine, calcium, iron, copper and magnesium), phenols (1–13%), vitamins, amino acids and small amounts of lipids (1–5%) to form a complex matrix [8–11]. Besides ECM polysaccharides, brown algae also produce laminarin (β-1,3-glucan) and mannitol [12] as storage carbohydrates.

Marine heterotrophic bacteria are crucial for algal biomass mineralization [13]. Macroalgae surfaces are constantly colonized by diverse bacterial communities with densities varying from 10^2^ to 10^7^ cells cm^−2^ of macroalgal tissue [14]. A fraction of these communities, mainly *Bacteroidetes*, *Gammaproteobacteria*, *Verrucomicrobia* and *Planctomycetes*, can degrade this complex biomass, showing abilities to hydrolyze purified high molecular weight algal compounds using a considerable enzymatic arsenal [15–18]. Over the last 20 years, many studies investigated the algal polysaccharide-processing capabilities of marine heterotrophic bacteria [19], deciphering new catabolic pathways and unraveling the role of carbohydrate active enzymes (CAZymes, http://www.cazy.org, [20]) including glycoside hydrolases (GHs), polysaccharide lyases (PLs) or carbohydrate esterases (CEs), and sulfatases (http://abims.sb-roscoff.fr/sulfatlas/, [21]). In *Bacteroidetes*, CAZymes are usually organized within clusters of co-regulated genes involved in carbohydrate binding, hydrolysis and transport, known as polysaccharide utilization loci (PULs). The regulations of these PULs during purified algal substrate degradation were recently studied in a few transcriptome-wide analyses, for both cultivated marine bacteria [22–26] and natural seawater bacterial communities [27]. However, using unique substrates does not reflect the complexity of the responses that might occur during the degradation of intact algal biomass. The term “pioneer” has been previously introduced to describe bacteria specialized in the breakdown of intact polysaccharides and release of degradation products that can fuel so-called scavenger bacteria [19, 28–30]. In nature, some pioneers should therefore be able to initiate algal tissue degradation and expose new substrate niches for other community members. Yet, the metabolic strategies of such algae-degrading pioneer bacteria have been seldom studied despite their crucial ecological relevance. To our knowledge, no previous work investigated the mechanisms involved in the degradation of fresh macroalgae, hindering our understanding of algal biomass recycling in coastal habitats. To date information on the mechanisms involved in raw algal material assimilation is scarce. “*Bacillus weihaiensis*” Alg07^T^ and *Bacillus* sp. SYR4 grow with kelp and red algal powder, respectively [23, 31] and *Microbulbifer* CMC-5 grows with thallus pieces of the red alga *Gracilaria corticata* [32]. These studies suggested a successive use of the different brown algal polysaccharides contained in the algal ECM [23] and the release of degradation product in the medium [31, 32].

The genus *Zobellia* (*Flavobacteriaceae* family), frequently found associated with macroalgae [33–35], is composed of 15 validly described strains classified in 8 species [36–39]. Their genomes encode numerous CAZymes (263–336 genes representing from 6.4 to 7.6% of the coding sequences), and sulfatases [40–42]. Therefore, *Zobellia* spp. are considered as potent algal polysaccharide degraders. In particular, *Zobellia galactanivorans* Dsij^T^, isolated from a red macroalga [36, 43], is a model strain to study macroalgal polysaccharide utilization [44]. It allowed the discovery of many novel CAZymes and the description of new PULs targeting alginates [45–47], carrageenans [25], agars [48, 49], laminarin [50, 51], mix-linked glucan [52] and mannitol [53]. Its complete transcriptome analysis revealed common regulations triggered by polysaccharides from the same algal phylum [24]. *Z. galactanivorans* Dsij^T^ is also well equipped to cope with algal defenses and can accumulate iodine [42, 54, 55]. Moreover, a previous study suggested that *Z. galactanivorans* Dsij^T^ might act as pioneer bacteria by initiating the breakdown of the kelp *Laminaria digitata*, and demonstrated the crucial role of the alginate lyase AlyA1 in this process [56].

In this study, we aim to better understand the mechanisms controlling fresh macroalgae degradation. To tackle this issue, (i) the capacity of *Z. galactanivorans* Dsij^T^ to act as a pioneer in the utilization of fresh algal tissue and to favor the growth of non-pioneer taxa was tested to shed light on social bacterial behavior, (ii) the complete transcriptome of *Z. galactanivorans* Dsij^T^ was analyzed during the degradation of three brown macroalgae with distinct chemical composition and compared with purified sugars to decipher key genes and mechanisms specifically triggered with fresh tissues and (iii) the ability of *Z. galactanivorans* Dsij^T^ to degrade fresh algae tissues was compared with other *Zobellia* spp. to assess its singular role and hypothesize on potential genetic determinant in fresh macroalgae breakdown.

## Experimental procedure

### Purified substrates

Maltose (Sigma-Aldrich, St. Louis, MO, USA), alginate from *Laminaria digitata* (Danisco [ref. Grindsted FD176], Landerneau, France) and FCSP-enriched fraction (hereafter FCSPs) from *Ascophyllum nodosum* (Algues & Mer [HMWFSA15424, fraction >100 kDa], Ouessant, France) were tested for growth. Treatment of this commercial FCSP extract with the alginate lyase AlyA1 [46] followed by Carbohydrate-PAGE [57] revealed it contained alginate impurities. Colorimetric assays [58, 59] showed that uronic acids accounted for ~24% (w/w) of the FCSP extract. Based on previous measurements of 9% uronic acid content in pure FCSPs from *A. nodosum* [60], we therefore estimated the alginate contamination in the FCSP-enriched fraction to be ca. 15%. Alginate, agar (Sigma-Aldrich), kappa- (Goëmar, St. Malo, France) and iota-carrageenans (Danisco) were used for enzymatic assays.

### Strains

Eight *Zobellia* strains were used in this study (listed in Supplementary Table 1, together with previous results of their ability to use pure algal compounds [36–38]), as well as *Tenacibaculum aestuarii* SMK-4^T^ [61]. They were first grown in Zobell 2216 medium [62] at room temperature before inoculation in marine minimum medium (MMM) complemented with antibiotics to which all the tested strains are resistant (see supplementary methods for composition) and amended with 4 g l^−1^ maltose as the sole carbon source. Pre-cultures were centrifuged (3200 *g*, 10 min) and pellets washed twice in 1X saline solution. Cells were inoculated in microcosms at OD_600_ 0.05. For the co-culture experiment, *Zobellia galactanivorans* Dsij^T^ and *Tenacibaculum aestuarii* SMK-4^T^ were pre-cultured in Zobell 2216 medium only, as *T. aestuarii* does not grow in maltose-amended MMM.

### Macroalgae treatment

All algae were harvested at the Bloscon site (48°43′29.982″N, 03°58′8.27″W) in Roscoff (France) between May 2019 and March 2021 depending on the experiment. They were cut in pieces (ca. 2.5–3.5 cm^2^) with a sterile scalpel and immersed in 0.1% Triton in milli-Q water for 10 min followed by 1% iodine povidone in milli-Q water for 5 min to clean them from resident epibionts. Finally, algal pieces were rinsed in excess autoclaved seawater for 2 h, to minimize algal exudates and metabolites that could have been produced upon cutting.

### Microcosm set up and sampling

All experiments were performed at 20 °C in MMM supplemented with antibiotics and strains inoculated at an initial OD_600_ of 0.05. *Z. galactanivorans* Dsij^T^ was grown in 50 ml with 10 macroalgal pieces, either young *Laminaria digitata* (<20 cm), *Fucus serratus* or *Ascophyllum nodosum*. For comparison it was also grown in the same conditions using 4 g l^−1^ maltose, alginate or FCSPs. All conditions were performed in triplicates, except for *F. serratus* in duplicates. During the exponential phase (at 65 h for fresh algae, 24 h for maltose and 72 h for alginate and FCSPs), culture medium (10 ml) was retrieved on ice for RNA extraction from the free-living bacteria. On ice, 0.5 volume of killing buffer (20 mM Tris-HCl pH 7.5, 5 mM MgCl_2_, 20 mM NaN_3_) was added to the liquid samples and cell pellets were frozen in liquid nitrogen after centrifugation (3200 *g*, 10 min, 4 °C). In parallel, algae-attached cells were also recovered for RNA extraction, as detailed in Supplementary Methods.

To assess *Z. galactanivorans* growth when cultivated in contact or physically separated from algal tissues, incubations were performed in two-compartment vessels (100 ml each) with round bottom and a 65 mm flat edge opening (Witeg [ref. 0861050], Wertheim, Germany), separated by a 0.2 μm filter. Each compartment was filled with 30 ml of MMM and ten *L. digitata* pieces (meristem part, i.e. <15 cm from the base) were immersed in one.

Co-culture experiments were carried out in duplicates by inoculating *Z. galactanivorans* Dsij^T^ and *T. aestuarii* SMK-4^T^ in 50 ml with 15 pieces of the *L. digitata* meristem as the sole carbon source. Culture medium was collected during the degradation to monitor the growth of the two partners using sequential CARD-FISH.

For comparative physiology, the eight *Zobellia* strains were grown in 10 ml with three *L. digitata* pieces from the meristem part.

### RNA extraction and sequencing

Details of the protocols are available in Supplementary Methods. Briefly, free-living bacterial cells were lysed by incubation 5 min at 65 °C in lysis buffer (400 µl) and phenol (500 μl). After phenol-chloroform extraction, RNA was treated 1 h at 37 °C with 2 units of Turbo DNAse (ThermoFisher Scientific, Waltham, MA, USA), purified using NucleoSpin RNA Clean-up (Macherey-Nagel, Hoerdt, France) and eluted in 50 μl of nuclease-free water. This protocol was modified to extract RNA from algae-attached cells (see Supplementary Methods).

DNA contamination was checked by PCR with primers S-D-Bact-0341-b-S-17 and S-D- Bact-0785-a-A-21 targeting the 16S rRNA gene [63]. RNA was quantified using the Qubit RNA HS assay kit (ThermoFisher Scientific) and its integrity assessed on a Bioanalyzer 2100 (Agilent Technology, Santa Clara, CA, USA) with the Agilent RNA 6000 Pico kit.

Paired-end RNA sequencing (RNA-seq) was performed by the I2BC platform (UMR9198, CNRS, Gif-sur-Yvette) on a NextSeq instrument (Illumina, San Diego, CA, USA) using the NextSeq 500/550 High Output Kit v2 (75 cycles) after a Ribo-Zero ribosomal RNA depletion step. A total of 24 samples were sequenced (Supplementary Table 2).

### RNA-seq analysis

Demultiplexed and adapter-trimmed reads were processed with the Galaxy platform (https://galaxy.sb-roscoff.fr). After read quality filtering using Trimmomatic v0.38.0, transcripts were quantified using the pseudo-mapper Salmon v0.8.2 [64] with the *Z. galactanivorans* Dsij^T^ reference genome (retrieved from the MicroScope platform ([65], https://mage.genoscope.cns.fr), “zobellia_gal_DsiJT_v2”; Refseq NC_015844.1). Raw counts for individual samples were merged into a single expression matrix for downstream analysis. Raw and processed data were deposited under GEO accession number GSE189322. Cleaned reads were also mapped on the *Z. galactanivorans* Dsij^T^ reference genome using Bowtie2 ([66], Galaxy Version 2.3.2.2). The mean per nt coverage for the whole transcriptome was assessed using SAMtools v1.14 [67] (Supplementary Table 2). The mean per nt coverage and normalized read counts (after DESeq2 normalization) for three selected characterized house-keeping genes in *Z. galactanivorans* Dsij^T^ [68] are shown in Supplementary Table 3, and the coverage map for these three genes and for the well-characterized alginate PUL were visualized using the Integrative Genomics Viewer v2.11.9 [69] and shown in Supplementary Fig. 1. These data ensure the expression variability was not caused by low read coverage or promiscuous read mapping. Principal Component Analysis (PCA) and differential abundance analyses were performed on rlog-transformed data using *DESeq2* v1.26.0 package [70] in R v3.6.2 [71]. Genes displaying a log2 fold-change |log2FC | > 2 and a Bonferroni-adjusted *p* value < 0.05 were considered to be significantly differentially expressed. The upset plot was created using the *ComplexUpset* package [72, 73]. Hierarchical clustering was performed using the Ward’s minimum variance method [74]. Graphics were prepared using *ggplot2* [75].

### Enzymatic assays

One volume of 0.2 μm filtered supernatant from the microcosms was incubated with 9 volumes of 0.2% polysaccharide substrate at 28 °C overnight. Controls were prepared with boiled supernatants. The amount of reducing ends released was quantified using the ferricyanide assay [76]. For each sample, the activity measured in controls was subtracted. Finally, the mean value (*n* = 3) measured for the non-inoculated microcosms was subtracted. Significant differences (*p* < 0.05) from 0 were tested using t-tests.

## CARD-FISH

The *Zobellia*-specific probe ZOB137 was described in Brunet et al. 2021 [35] and the *Tenacibaculum*-specific probe TEN281 was designed in the same fashion (see Supplementary Methods).

Algal pieces and culture medium were fixed overnight at 4 °C with 2% paraformaldehyde. Free-living bacteria were harvested on a 0.2 μm polycarbonate membrane. Catalyzed reporter deposition-fluorescence in situ hybridization (CARD-FISH) was performed as described in [35] using the *Zobellia*-specific probe ZOB137 with helpers. For sequential CARD-FISH on co-culture medium, first hybridization and amplification were done using the probe ZOB137 and the fluorochrome Alexa546. HRPs were inactivated in 3% H_2_O_2_ (10 min), followed by a second hybridization and amplification with the probe TEN281 and the fluorochrome Alexa488. Cells on membrane were visualized with a Leica DMi8 epifluorescent microscope (oil objective 63X). Cells on algal tissues were detected with a Leica TCS SP8 confocal microscope (HC PL APO 63X/1.4 oil objective) using the 488 and 638 nm lasers to detect Alexa488 signal and algal autofluorescence signal, respectively. Z-stack images were collected using 1024 × 1024 scan format (0.29 μm thick layers, 400 Hz scan speed) and visualized using the surface channel mode of the 3D viewer module (Leica Las X software). Following sequential CARD-FISH, *Zobellia* and *Tenacibaculum* cell counts were processed manually from 5 different fields at each time.

### Comparative genomics

*Zobellia* genomes were screened for GHs, PLs, CEs and sulfatases using dbCAN2 [77] on the MicroScope platform. Homologs (>50% identity and >80% alignment) were searched for genes of interest using synteny results on MicroScope.

## Results

### *Zobellia galactanivorans* Dsij^T^ degrades fresh brown macroalgae tissues and benefits non-degrading bacteria

**Fig. 1:**
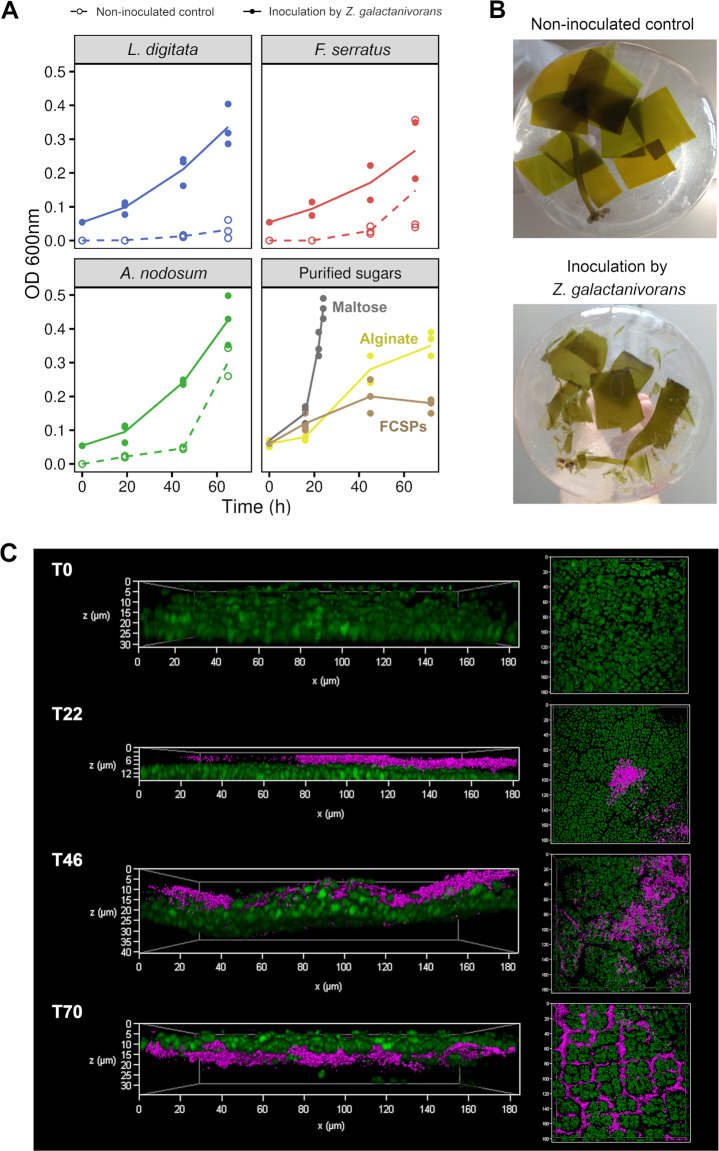
*Z. galactanivorans* Dsij^T^ is able to use different fresh brown macroalgae for its growth. **A** Growth of *Z. galactanivorans* with either macroalgae pieces (*Laminaria digitata*, *Fucus serratus* and *Ascophyllum nodosum*) or purified sugars (maltose, alginate and FCSPs). Individual points for replicate experiments are shown. Lines are means of independent replicates (*n* = 2 or *n* = 3). **B** Photographs showing the integrity of the *L. digitata* tissues after 65 h. **C** *L. digitata* tissues colonization by *Z. galactanivorans* during the degradation. Micrographs are overlay of the CARD–FISH signal (magenta, *Zobellia*-specific probe with Alexa488 as the reporter signal) and the algal autofluorescence (green) and were obtained with the surface channel mode of the 3D viewer. For the different times, transversal views are shown on the left and top views on the right. The non-fluorescent gap between the bacterial cells and the algal cells likely represents the mucilage coat of *L. digitata*. The absence of algal autofluorescence signal below 25–30 µm is the result of its rapid decrease in intensity as we move away from the coverslip.

*Z. galactanivorans* growth was tested with three brown macroalgae from two different orders and with distinct chemical composition, *Laminaria digitata* (order Laminariales), *Fucus serratus* and *Ascophyllum nodosum* (order Fucales), as the sole carbon and energy source. Growth was detected with the three algal species (OD ≈ 0.2–0.5, Fig. 1A), with tissue bleaching and damages only visible on *L. digitata* pieces after 65 h (Fig. 1B). *Zobellia*-specific CARD-FISH assays revealed that even if antibiotic-resistant resident epibionts grew in the non-inoculated controls containing *A. nodosum* and *F. serratus* (one replicate), most of the bacterial biomass after 65 h in the *Zobellia*-inoculated microcosms was *Zobellia* cells (Supplementary Fig. 2).

**Fig. 2:**
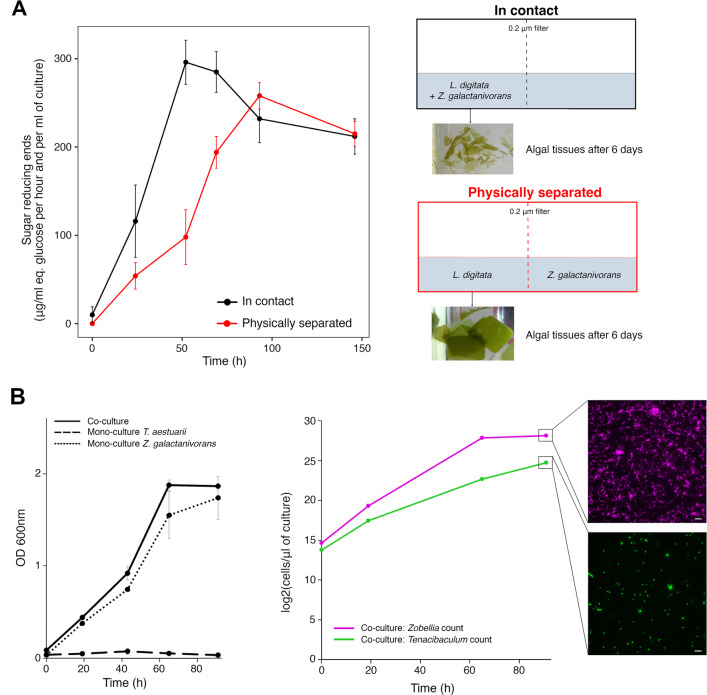
Macroalgae degradation by *Z. galactanivorans* Dsij^T^ allows the growth of other taxa. **A** Alginolytic activity of the enzymes secreted when *Z. galactanivorans* was grown in contact with *L. digitata* (black) or separated from *L. digitata* by a 0.2 µm filter (red). The activity was measured in each compartment (left and right) and summed. Values are mean ± s.d. (*n* = 3). **B** Cooperative interactions between *Z. galactanivorans* Dsij^T^ and *T. aestuarii* SMK-4^T^. Left: Co-culture and monoculture growth curve with *L. digitata* as sole carbon source. Values are replicate mean ± s.d. (*n* = 2). Right: *Zobellia* (magenta) and *Tenacibaculum* (green) cell concentration during the co-culture experiment. Cell counts were performed from 5 different microscopic fields of micrographs obtained after sequential CARD-FISH on one replicate. One microscopic field from the sample collected at 91 h is shown, showing either the *Zobellia* (magenta signal) or the *Tenacibaculum* (green signal) signal. Scale = 10 μm.

CARD-FISH assays on *L. digitata* tissues showed gradual tissue colonization by *Z. galactanivorans*, from cell patches at the surface of the *L. digitata* mucilage coat to deeper penetration within the tissue invading the intercellular space (Fig. 1C). To assess if algal degradation requires cell attachment, *Z. galactanivorans* was grown either in contact or physically separated from algal pieces (Fig. 2A). After 6 days, algal tissues were visually starting to decompose when bacteria were separated from algae, although to a lesser extent compared to the “contact” condition. Furthermore, extracellular alginolytic activity increased even without physical bacteria/algae contact and reached similar levels to that observed in the “contact” condition after 90 h. We further tested if this degradation behavior of *Z. galactanivorans* could lead to cooperative interactions with non-degrading bacteria. As a test case, we used the flavobacterium *Tenacibaculum aestuarii* that was not able to feed on fresh *L. digitata* pieces or initiate its degradation (Fig. 2B). When *T. aestuarii* was cultivated together with *Z. galactanivorans*, the optical density of the co-culture was between 7 and 23% higher than in the *Z. galactanivorans* monoculture. Sequential CARD-FISH analyses indicated an exponential growth of *T. aestuarii* during algal degradation in the co-culture microcosms, reaching 2.8 × 10^7^ cells µl^−1^ after 91 h. *T. aestuarii* density was ~10 times lower and delayed compared to *Z. galactanivorans*. This delay suggests that *T. aestuarii* is able to grow after the accumulation of degradation products or secondary metabolites in the medium.

### The transcriptomic profile of free-living bacteria shifts during fresh macroalgae degradation

**Fig. 3:**
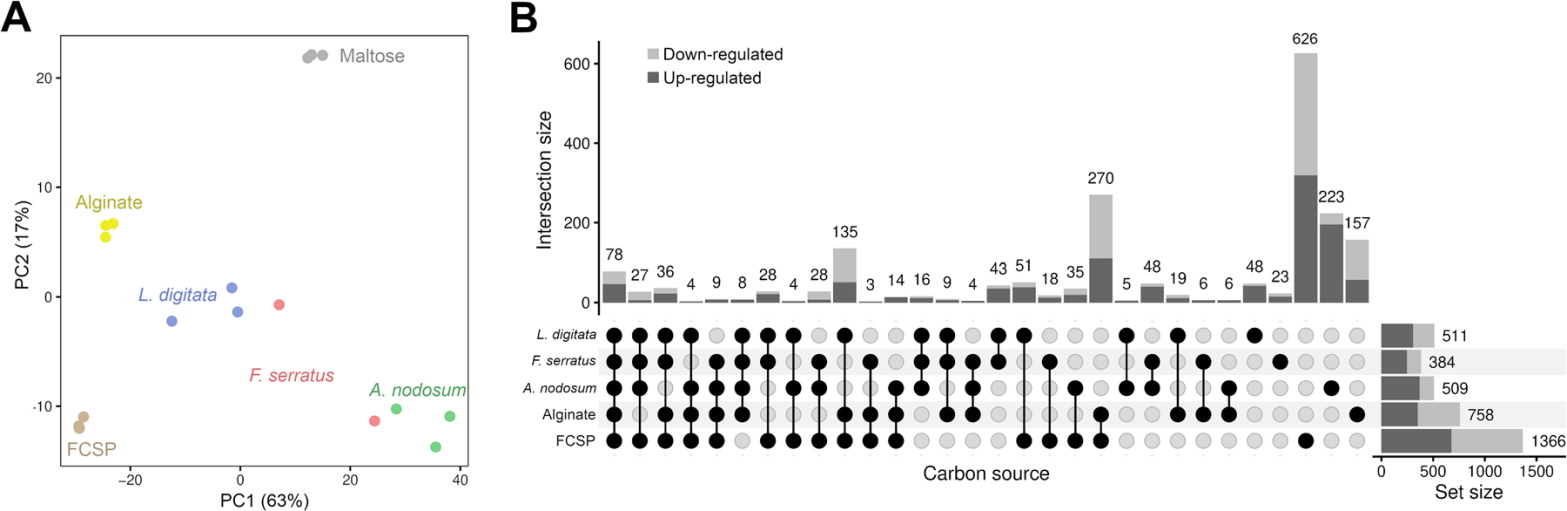
General features of the transcriptomic responses occurring in free-living *Z. galactanivorans* Dsij^T^ during growth with macroalgae. **A** Principal Component Analysis of the gene expression. **B** Upset plot of the differentially expressed genes with maltose as the control condition (Bonferroni-adjusted *p* value < 0.05 and |log2FC | > 2). Set size represents the total amount of genes regulated in each condition.

*Z. galactanivorans* Dsij^T^ transcriptome of free-living cells obtained during macroalgal degradation was compared to the responses occurring with a disaccharide, maltose, and with purified brown algal polysaccharides, alginate and FCSPs. Between 44 and 93% of the sequenced reads from free-living bacteria grown with macroalgae mapped on the genome of *Z. galactanivorans* Dsij^T^ (Supplementary Table 2). Multivariate analysis separated samples according to carbon source (Fig. 3A). Transcriptomes of cells grown with *L. digitata* were closer to that obtained with alginate or FCSPs compared to *A. nodosum* or *F. serratus*.

Differential abundance analysis revealed 1117 and 864 genes up- and down-regulated with at least one substrate, using maltose as control (Supplementary Table 4). Among them, 56% (628 upregulated genes) and 52% (449 downregulated genes) showed substrate-specific regulations (Fig. 3B). In particular, half of the genes regulated with *A. nodosum* and FCSPs were not differentially expressed in any other conditions. *L. digitata* was the algal species inducing the highest number of regulated genes shared with at least one polysaccharide (399, 254 and 217 genes with *L. digitata*, *F. serratus* and *A. nodosum* respectively). More regulations were shared between *L. digitata* and *F. serratus* (116 genes) than *F. serratus* and *A. nodosum* (89 genes) or *L. digitata* and *A. nodosum* (13 genes). Finally, a core set of 70 upregulated and 59 down-regulated genes responded to the three macroalgae.

### Carbohydrate catabolism-related genes

**Fig. 4:**
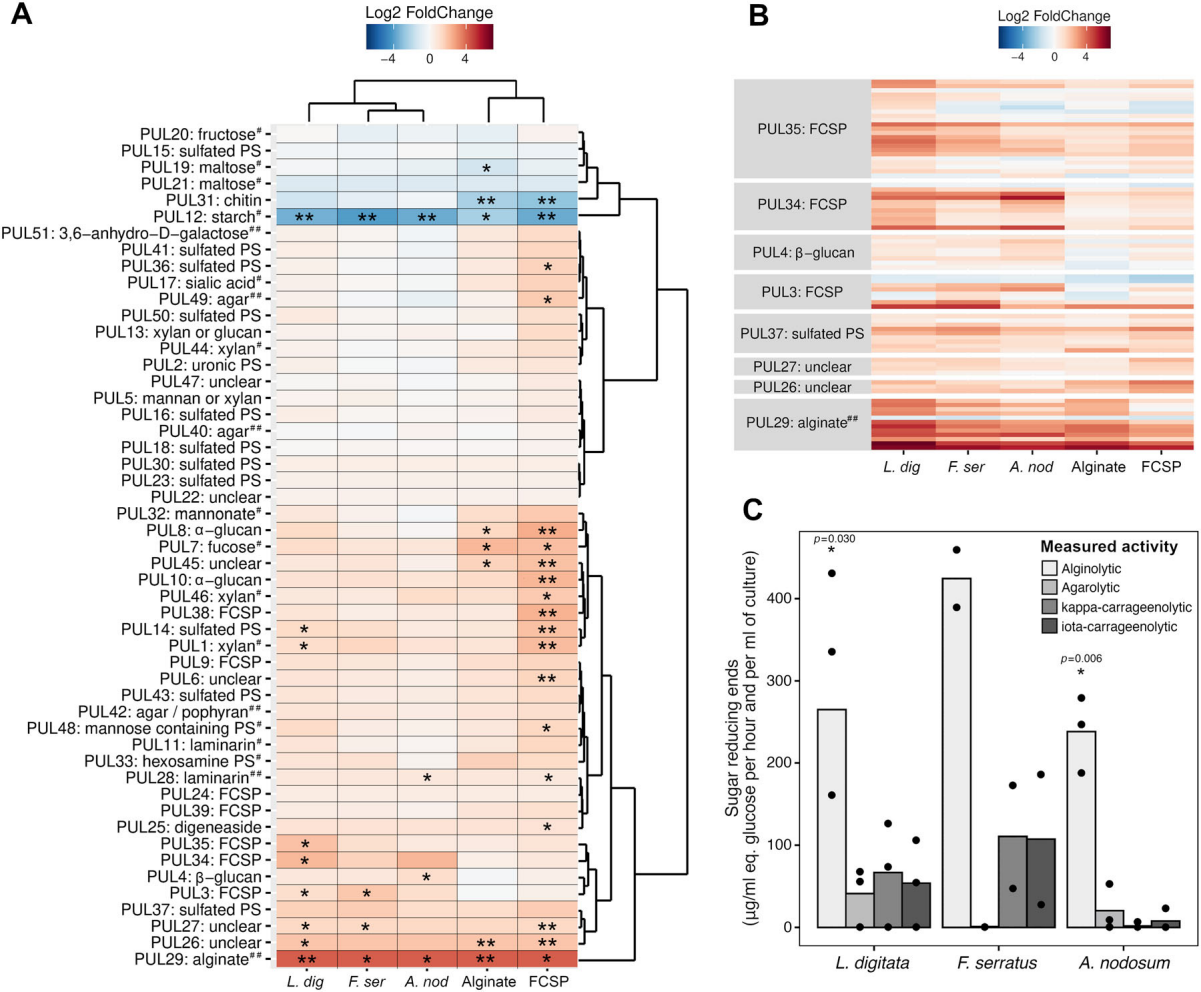
Specific upregulation of pathways involved in brown algae carbohydrate catabolism during the utilization of fresh brown algal tissues. **A** Heatmap of the 51 PULs identified in the genome of *Z. galactanivorans* Dsij^T^. PUL 1 to 50 were identified during the annotation of the *Z. galactanivorans* Dsij^T^ genome by the presence of the *susCD*-like pair, their boundaries are based on bioinformatic predictions (Supplementary Table S3 in [42]). PUL51 targeting 3,6-anhydro-D-galactose and involved in carrageenan catabolism (but lacking the *susCD*-like pair) was further described [25]. For each PUL, the mean log2FC of all genes is represented, taking maltose as a control condition. Carbon sources and PULs were arranged according to a hierarchical clustering analysis (Ward’s method). A PUL was considered regulated (induced in red, repressed in blue) if more than 50% of the genes were significantly differentially expressed (*) and strongly regulated if more than 80% of the genes were significantly differentially expressed (**). Putative substrates targeted by the PULs are indicated. Hash signs denote PULs biochemically characterized previously in *Z. galactanivorans* (##) or in another organism (#). **B** Heatmap representing the log2FC of individual genes contained in the PULs induced with macroalgae and which clustered together in **A**. **C** Activity of extracellular polysaccharidases collected in the microcosms containing macroalgae after 65 h. The mean value measured in the uninoculated controls was subtracted from each value. Bars are means of independent replicates (*n* = 2 or 3) shown as individual points. Significant difference from zero was tested when *n* = 3 (*t*-test; *p < 0.05). *L. dig*: *Laminaria digitata*; *F. ser*: *Fucus serratus*; *A. nod*: *Ascophyllum nodosum*; FCSP: fucose-containing sulfated polysaccharide; PS: Polysaccharide.

Hierarchical clustering of expression data of the 51 identified PULs in the *Z. galactanivorans* Dsij^T^ genome revealed that PULs predicted to target brown algal polysaccharides grouped together (Fig. 4A) and were significantly induced with macroalgae. In particular, the alginate-specific PUL29 was significantly overexpressed in all conditions compared with maltose (mean log2FC of 4) and the highest expression was observed with *L. digitata* (Fig. 4B, Supplementary Fig. 3). Some PULs were exclusively triggered by macroalgae: PUL34 and 35, likely targeting FCSPs (as they encode sulfatases and fucosidases), were significantly triggered by *L. digitata*, PUL4 targeting β-glucan responded to *A. nodosum* and the FCSP PUL3 was induced by both *L. digitata* and *F. serratus*. PUL26 and 27, whose function remains unclear, were both induced by *L. digitata* and FCSPs, as well as by alginate for PUL26 and *F. serratus* for PUL27. FCSPs also induced the expression of 14 PULs outside the described cluster, encompassing a large diversity of targeted substrate (notably β- and α-glucan, sulfated polysaccharides, xylan, unclear substrate). No PUL known to target red algal polysaccharides (e.g. PUL40, 42, 49 or 51) clustered with this set of overexpressed PULs, suggesting a specific induction of brown algal polysaccharide degradation mechanisms in the presence of brown algal tissues. The measured activity of secreted polysaccharidases corroborates this observation (Fig. 4C), as only the alginolytic activity was significantly higher when *Z. galactanivorans* was grown on macroalgae compared with the non-inoculated control (*t*-test, *p* < 0.05).

On the other hand, PULs targeting simple sugars (maltose and fructose) or polysaccharides absent from brown algae (starch and chitin) were repressed with macroalgae and purified polysaccharides (Fig. 4A). The starch PUL12 was strongly under-expressed in all conditions while the chitin PUL31 showed a significant repression only with algal polysaccharides.

### Specific induction with fresh algal tissues

**Fig. 5:**
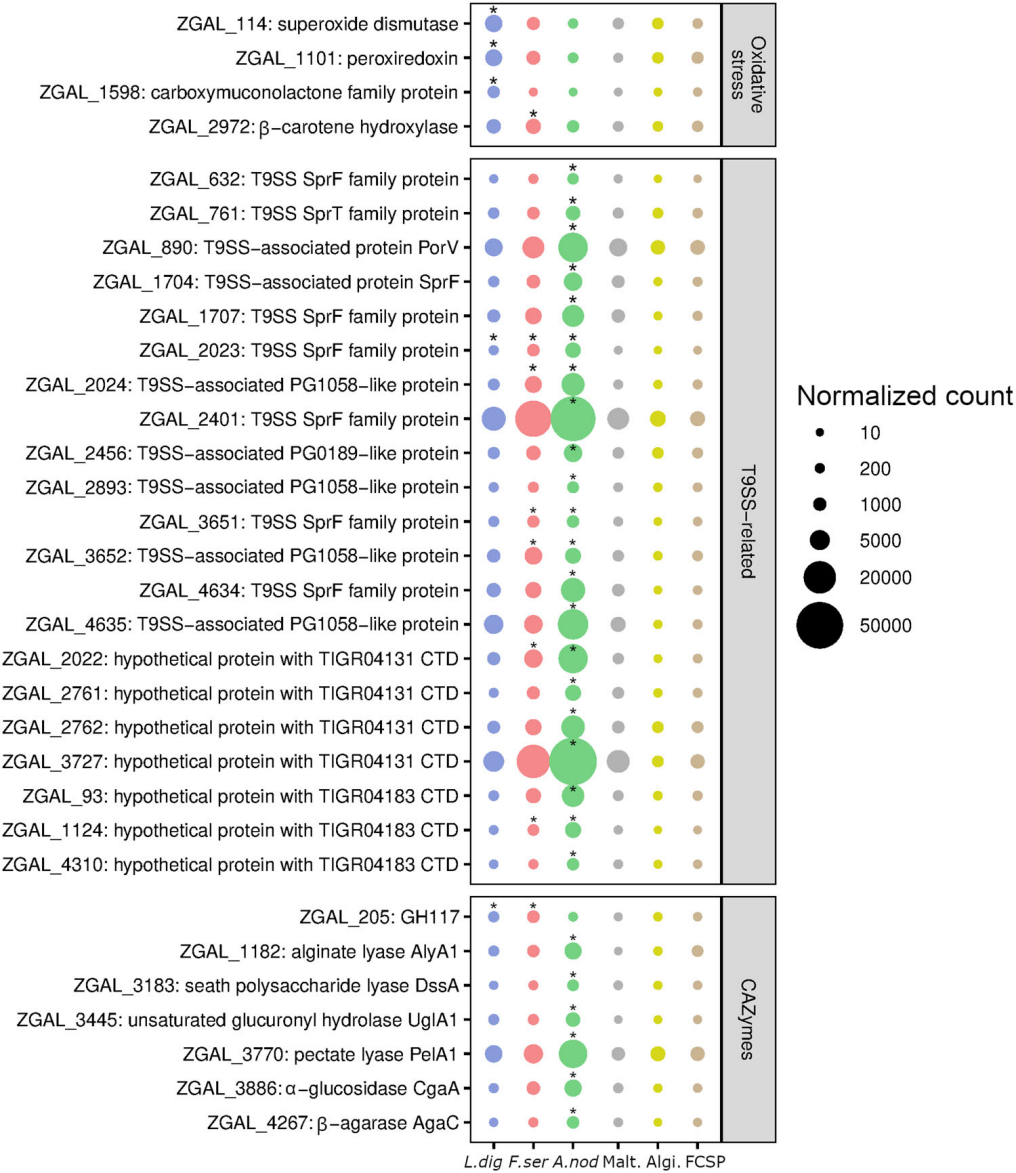
Genes related to oxidative stress response and T9SS are specifically induced by fresh tissue. Mean expression values (*n* = 3, except for *F. serratus* *n* = 2) of selected genes significantly triggered (*) with at least one macroalgae compared to both the purified polysaccharides and maltose (see Supplementary Table 4). *L. dig*: *Laminaria digitata*; *F. ser*: *Fucus serratus*; *A. nod*: *Ascophyllum nodosum*; Malt.: Maltose; Algi.: Alginate; FCSP: fucose-containing sulfated polysaccharide.

To unravel pathways specifically governing the degradation of fresh macroalgal biomass, we further focused on genes upregulated with at least one macroalgal species compared to maltose and purified polysaccharides. We detected 41, 59 and 189 genes following this pattern with *L. digitata*, *F. serratus* or *A. nodosum*, respectively (Supplementary Table 5). It included few CAZyme-encoding genes (Fig. 5), notably two genes within putative FCSP PULs (*zgal_205* [GH117 in PUL3] and *zgal_3445* [GH88 in PUL34]). Other polysaccharidase genes outside classical PUL structures were induced with *A. nodosum*, such as *alyA1* (*zgal_1182*, alginate lyase PL7), *cgaA* (*zgal_3886*, glucan 1,4-α-glucosidase GH15), *agaC* (*zgal_4267*, β-agarase GH16), *pelA1* (*zgal_3770*, pectate lyase PL1) and *dssA* (*zgal_3183*, sheath polysaccharide lyase PL9). GT2 (*zgal_2991*, *4154*) and GT4 (*zgal_2990*, *3759*) were also triggered with macroalgae. Additionally, many genes linked to oxidative stress responses and Type IX secretion systems (T9SS) were specifically induced with macroalgae (Fig. 5). A large gene cluster (*zgal_1071-1105)* notably encoding three oxidoreductases, a DNA topoisomerase and a peroxiredoxin was upregulated with *L. digitata* and *F. serratus*. Other genes encoding antioxidant proteins were triggered, especially on *L. digitata*, such as the superoxide dismutase SodC (ZGAL_114) or a β-carotene hydroxylase (ZGAL_2972), as well as a carboxymuconolactone decarboxylase family protein (ZGAL_1598) which includes enzyme involved in antioxidant defense [78]. Two catalases (ZGAL_1427 and ZGAL_3559) were induced in the presence of *L. digitata* and *F. serratus* in comparison to maltose and alginate (Supplementary Table 5). Despite the poor sequencing depth of RNA extracted from algae-attached cells (Supplementary Table 2), the induction of stress resistance mechanisms tended to be even more pronounced in algae-attached bacteria compared with the free-living ones, especially through the expression of chaperones (Supplementary Fig. 4).

Several genes predicted to encode T9SS components were significantly induced during macroalga degradation, in particular with *A. nodosum* (14 out of 33 genes identified in the genome, against 1 and 5 with *L. digitata* and *F. serratus* respectively) (Fig. 5). They include particularly genes encoding SprF family proteins and T9SS-associated PG1058-like proteins. In addition, 7 unknown proteins containing a conserved C-terminal domain (CTD) from families TIGR04131 (gliding motility - ZGAL_2022, 2761, 2762, 3727) and TIGR04183 (Por secretion system - ZGAL_93, 1124, 4310) were triggered. These CTDs are typical of cargo proteins secreted by the T9SS.

### Comparative physiology and genomics of fresh macroalga degradation by *Zobellia*

**Fig. 6:**
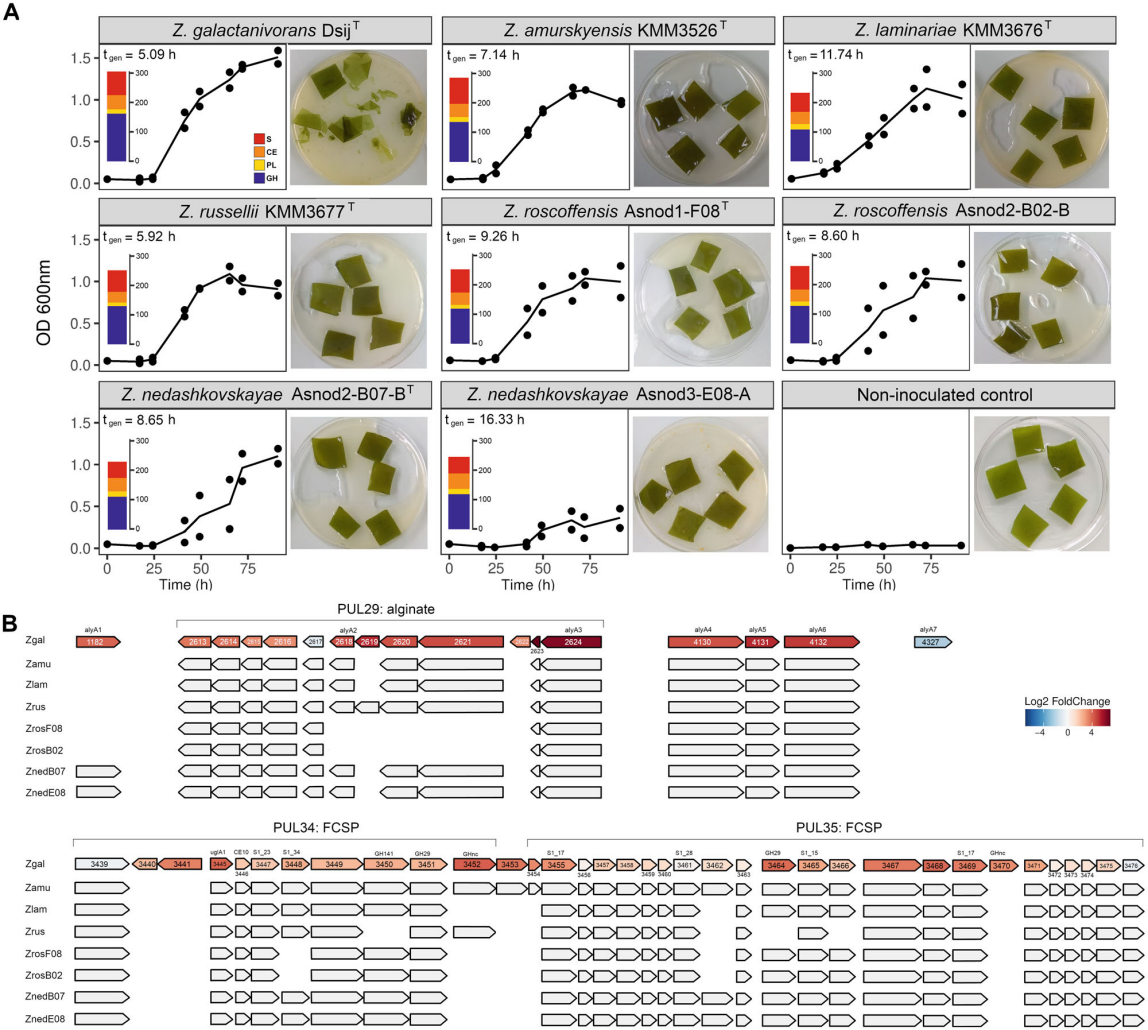
All *Zobellia* spp. use fresh *L. digitata* but *Z. galactanivorans* Dsij^T^ displays the highest tissue breakdown capacity. **A** Growth of eight *Zobellia* strains with *L. digitata* pieces (meristem of adult individuals). The generation time t_gen_ is indicated for each strain as well as the number of glycoside hydrolases (GH, blue), polysaccharides lyases (PL, yellow), carbohydrate esterases (CE, orange) and sulfatases (S, red) predicted in their genome (dbCAN search on the MaGe platform). Individual points for duplicate experiments are shown. Lines are means of independent replicates (*n* = 2 or *n* = 3). **B** Comparison of genomic loci among the eight *Zobellia* strains. For *Z. galactanivorans*, genes were colored according to their expression log2FC for the comparison *L. digitata* vs. maltose. Gene ID is indicated inside arrows and CAZymes and sulfatases are specified above. Top: genes involved in the alginate-utilization system. Bottom: genes contained in putative FCSP PUL34 and 35. Zgal: *Z. galactanivorans* Dsij^T^; Zamu: *Z. amurskyensis* KMM 3526 ^T^; Zlam: *Z. laminariae* KMM 3676 ^T^; Zrus: *Z. russellii* KMM 3677 ^T^; ZrosF08: *Z. roscoffensis* Asnod1-F08^T^; ZrosB02: *Z. roscoffensis* Asnod2-B02-B; ZnedB07: *Z. nedashkovskayae* Asnod2-B07-B^T^; ZnedE08: *Z. nedashkovskayae* Asnod3-E08-A.

The degrading abilities of other members of the genus *Zobellia* were investigated (Fig. 6A). All tested *Zobellia* strains used fresh *L. digitata* tissues for their growth. *Z. galactanivorans* Dsij^T^ had the highest final cell density (OD_600_ = 1.5) and shortest generation time (5.09 h). *Z. nedashkovskayae* Asnod3-E08-A formed cell aggregates that biased OD_600_ readings, likely explaining the apparent limited growth (final OD_600_ = 0.4) and long generation time (t_gen_ = 16.33 h). Other strains showed intermediate behaviors (OD_600_ ≈ 1, 5.92 < t_gen_ < 11.74 h). These growth differences were reflected in the final aspect of macroalgal pieces. Only *Z. galactanivorans* Dsij^T^ completely broke down algal tissues after 91 h. Both *Z. nedashkovskayae* strains caused limited algal peeling and breakdown at the corners of the pieces., No visible trace of degradation was detected for other strains. A strong negative correlation was found between the number of GHs and the generation time (Spearman, rho = −0.90; *p* = 0.006) (Fig. 6A, Supplementary Table 6). Twenty-two out of the 305 genes upregulated by *Z. galactanivorans* Dsij^T^ with *L. digitata* compared to maltose had no homologs in the genome of the seven other *Zobellia* strains (Supplementary Table 7). They include two GHs, *zgal_3349* (GH20 in PUL33) and *zgal_3470* (GHnc in PUL35), and a susCD-like pair (*zgal_3440, 3441*) in PUL34. Other upregulated genes within the FCSP PUL34/35 are not conserved in all *Zobellia* strains (Fig. 6B). Likewise, several alginolytic genes were not conserved across the genus, especially in the two *Z. roscoffensis* strains that lack 7 of them. *zgal_1182* and *zgal_4327*, encoding the extracellular endo-alginate lyases AlyA1 and AlyA7 respectively, were not conserved in the other strains (*zgal_4327*) or only found in the *Z. nedashkovskayae* strains (*zgal_1182*). Two other genes related to carbohydrate assimilation (*zgal_334* and *zgal_2296* encoding a GHnc and a lipoprotein with CBM22, respectively) are missing in five strains (Supplementary Table 7). *zgal_334* neighbors genes encoding sulfatases, fucosidases and PLs and might belong to a FCSP-targeting cluster (absent from the 51 identified PULs as the pair *susCD*-like is absent).

## Discussion

### *Zobellia galactanivorans* Dsij^T^: a sharing pioneer role during brown macroalgae utilization

By degrading macroalgae, marine heterotrophic bacteria are central to nutrient cycling in coastal habitats. The ecological strategies of different functional guilds not equally equipped to process biomass were recently conceptualized [19, 28, 29]. First, pioneer bacteria degrade complex organic matter by producing specific hydrolytic enzymes. The hydrolysate can then fuel other bacteria called exploiters or scavengers, which cannot feed on intact substrates. Such cooperative interactions were previously characterized during alginate [79] or chitin [80, 81] assimilation. Hence, in nature pioneer bacteria likely control the initial attack on fresh macroalgae, a hitherto rarely studied process that cannot be fully deciphered when using purified polysaccharides or crushed algae. Here, we showed that *Z. galactanivorans* Dsij^T^ uses fresh brown algal tissues for its growth, highlighting its pioneer role in algal biomass recycling. Similar growth rates were observed with three brown algal species, and *Z. galactanivorans* completely broke down *L. digitata* tissues. Transcriptomes obtained with *L. digitata* were closest to that with alginate and FCSP, suggesting a greater capacity to access and digest ECM polysaccharides within the *L. digitata* tissues compared to *A. nodosum* and *F. serratus*. The limited degradation of Fucales tissues might originate from their higher phlorotannin content [82], possibly inhibiting CAZymes [83]. In addition, *A. nodosum* induced a wider cellular response with many specific regulations. This might partly be due to the growth of antibiotic-resistant epiphytic bacteria that could have affected *Z. galactanivorans* behavior or to its much thicker and rigid thallus. Furthermore, *A. nodosum* is associated with various symbionts, especially the obligate endophytic fungus *Mycophycias ascophylli* [84] that secretes compounds potentially preventing tissue grazing and/or offering additional substrate niches.

We showed that although *Z. galactanivorans* can colonize *L. digitata*, it does not require a physical contact to initiate algal breakdown. This suggests a crucial role for secreted enzymes during the first stages of the degradation, in line with the measured extracellular alginolytic activity. Constitutively expressed extracellular enzymes, such as the alginate lyases AlyA1 and AlyA7 acting as sentry enzymes, would rapidly release diffusible degradation products, allowing remote substrate sensing [45]. We previously showed that when grown with purified alginate or algal tissues, *Z. galactanivorans* accumulates low molecular weight (LMW) alginate oligosaccharides that act as effectors for the expression of the alginolytic PUL [45, 56]. The release of degradation products could possibly govern cooperative interactions between *Z. galactanivorans* and other taxa. We specifically demonstrated here that *Z. galactanivorans* was able to support the growth of a *Tenacibaculum* species during algal tissue utilization, as first suggested in a previous study [56]. Therefore, we propose that *Z. galactanivorans* would behave as a “sharing” pioneer: by initiating algal breakdown, it would provide degradation products as public goods to opportunist taxa, contrary to “selfish” pioneers which sequester LMW products by producing essentially surface-associated hydrolytic enzymes with minor loss of hydrolysate to the medium [85, 86]. *Tenacibaculum* spp. were regularly reported on macroalgae [87], although their role in macroalgae degradation remains unclear. Members of the genus *Tenacibaculum* were identified as active incorporators of carbon from alginate within natural seawater communities [88]. The number of CAZymes is highly variable within the genus (between 34 and 102 for the 8 *Tenacibaculum* genomes available in the CAZy database) but is largely below the 221 CAZymes found in *Z. galactanivorans* Dsij^T^ [20]. *Tenacibaculum* spp. might then lack key genetic determinants to initiate the algal breakdown but are likely able to use processed algal compounds. On the other hand, the benefits of cooperative interactions for sharing pioneers remain elusive. Indeed, studies on chitin degradation by marine bacterial communities suggest that the presence of non-degrading organisms decreases degradation rates and overall productivity, possibly due to competition for space and/or nutrients [89, 90]. Yet, the benefits of cooperative interactions might be more relevant in limiting conditions, if scavengers produce one or several public goods (e.g. vitamins, siderophores) to be used by pioneers [91]. Additionally, scavengers might hinder the growth of other competing microorganisms that may have negative interactions with pioneers.

We further showed that the pioneer behavior can be strain-specific within the alga-associated genus *Zobellia*. All *Zobellia* spp. tested successfully grew with fresh *L. digitata* but without causing pronounced tissues damages as observed with *Z. galactanivorans*. Their catabolic profiles (Supplementary Table 1) indicate different growth capacities with purified brown algal sugars. For example, *Z. roscoffensis* strains and *Z. laminariae* KMM 3676 ^T^ display limited or no abilities to use alginate, FCSPs and laminarin for their growth. Hence, with macroalgae, they likely did not use these complex polysaccharides but rather fed on soluble algal exudates (e.g. mannitol). At least some of the tested *Zobellia* strains are known to co-occur in nature: *Z. roscoffensis* and *Z. nedashkovskayae* strains were isolated simultaneously from brown algae in Roscoff, and *Z. galactanivorans* Dsij^T^ was retrieved from the same location 30 years earlier. Therefore, different *Zobellia* species likely stably coexist at the surface of algae and their contrasted behavior towards fresh algal biomass degradation suggests they might not compete for the same resources, thus occupying different ecological niches. Some species, especially *Z. galactanivorans* Dsij^T^, would be well adapted to breakdown fresh brown algae, while others would exploit soluble carbon sources such as dissolved polysaccharides, algal exudates (e.g. mannitol), degradation products (oligosaccharides, secondary metabolites) or non-carbohydrate cell wall compounds (e.g. proteins). Comparative genomics suggested that CAZyme content influences the strain capacity to use and break down fresh algal tissues. The highest number of CAZymes in *Z. galactanivorans* Dsij^T^ could explain its greater success at degrading algae. In particular, some *Zobellia* strains lack homologs of overexpressed genes contained in *L. digitata-*induced PULs targeting alginate or FCSPs. For example, *alyA1* homologs were only found in the two other strains that caused visible algal damage (*Z. nedashkovskayae* Asnod2-B07-B^T^ and Asnod3-E08-A). Accordingly, *alyA1* is known to have a crucial role in initiating algae breakdown [56]. Such genes would therefore represent potential genetic determinants of pioneer bacteria. Yet, *Z. galactanivorans* Dsij^T^ lacks key genes to use some specific algal compounds, including particular FCSP structures (e.g. FCSP from *Pelvetia canaliculata*) or ulvans from green algae [42]. When faced with such compounds, *Z. galactanivorans* Dsij^T^ might therefore benefit from other highly specialized taxa possessing complete degradation pathways, such as members of the verrucomicrobium genus ‘*Lentimonas*’ [92] or the flavobacterium *Formosa agariphila* KMM 3901^T^ [93] for FCSP and ulvan degradation respectively. This and other differences in gene content or regulatory programs between co-occurring strains could also explain the maintenance of a diverse pioneer bacteria population potentially acting as a consortium towards the complete breakdown of macroalgae.

### Deciphering the metabolic mechanisms involved in fresh tissue breakdown, including new catabolic pathways

Regardless of the algal species, the well-characterized alginolytic PUL29 was the most induced among all PULs. Alginate is the most abundant polysaccharide in brown algal ECM and likely the most accessible as it embeds the cellulose-FCSP network [11]. This PUL was particularly triggered with *L. digitata*, likely reflecting the higher alginate content in this species [2] and/or easier substrate accessibility. Furthermore, several uncharacterized PULs were triggered with macroalgae. In particular, three out of the seven predicted FCSP PULs were significantly upregulated with macroalgae but not with extracted *A. nodosum* FCSPs, and to various degrees depending on algal species. This suggests different substrate specificities, consistent with the large structural diversity of FCSPs and cross-linkage to other compounds [4, 7, 94] that might not be equally extracted during purification. By preserving the original polysaccharide structure and environment, the study of fresh macroalga degradation may therefore be a more effective way to reveal specific genes crucial for macroalgae breakdown by pioneer bacteria but undetectable when using purified polysaccharides.

By contrast to alginate- and FCSP-targeting PULs, the characterized laminarin PUL11 and PUL28 were poorly regulated with the three algae. An uncharacterized β-glucan PUL4 was significantly induced only with *A. nodosum*, and also found triggered with purified laminarin in a previous study [24]. As raised above, the presence of endosymbionts in *A. nodosum* could result in specific laminarin structures that might be targeted by PUL4. The absence of induction of typical laminarin PULs with macroalgae might also indicate that *Z. galactanivorans* Dsij^T^ first uses ECM polysaccharides and later access intracellular storage polysaccharides. Such a prioritization of multiple substrates within algal material was previously observed for *Bacillus weihaiensis* Alg07^T^ grown on algal powder [23]. Koch et al. [26] showed that *Alteromonas macleodii* 83-1 prioritized laminarin over alginate and pectin when grown on a mixture of purified polysaccharides. Thus, prioritization might differ between bacterial strains and whether substrates are under soluble form or within algal tissues, underlining the importance to consider intact macroalgae to understand the pioneer behavior. Furthermore, future time-resolved transcriptome analyses could inform on regulations at different degradation stages and help decipher prioritization effects.

Besides carbohydrate utilization, our approach unveiled several traits specifically induced upon macroalgal degradation and potentially linked to the pioneer behavior, including the resistance to algal defense and T9SS. One of the algal defense mechanisms is the production of reactive oxygen species (ROS), which in *L. digitata* is partly induced by endogenous elicitors (i.e. oligo-alginates) derived from the degradation of their own cell wall [95]. Breakdown of *L. digitata* tissues by *Z. galactanivorans* likely produced large amounts of elicitors, triggering a massive accumulation of ROS in the closed microcosm setup, in line with the strong induction of genes encoding ROS-detoxifying enzymes in this condition. In contrast, *A. nodosum* and *F. serratus* do not respond to the addition of endogenous elicitors [96], potentially explaining the lower induction of antioxidant pathways in *Z. galactanivorans* Dsij^T^ with these algae. To our knowledge, this is the first time ROS detoxification is shown as an important component of macroalgae degradation by marine bacteria. It is reminiscent of previous results showing the induction of oxidative stress responses in plant-associated terrestrial bacteria [97]. Another algal defense response is the emission of halogenated compounds. One vanadium-dependent iodoperoxidase (vIPO3) and a haloacid dehalogenase (HAD, [55]) were significantly upregulated with *A. nodosum* compared to alginate and maltose respectively. HAD expression was also 3-fold higher with *L. digitata* and *F. serratus* compared to maltose, although large variations precluded significance. Overall, our results suggest that pioneer bacteria might have evolved to cope with increasing stress levels upon algal degradation. Such metabolization of toxic compounds might also be a hitherto overlooked additional benefit that sharing pioneer bacteria provide to less stress-resistant scavengers. For example, Grigorian et al., 2021 [55] revealed that *Tenacibaculum* is one of the two genera within the *Flavobacteriaceae* family lacking Type II haloacid dehalogenase and that the growth of *Tenacibaculum* strains was inhibited by iodo and bromoacetic acid, while *Z. galactanivorans* Dsij^T^ was more tolerant. Hence, besides the sharing of degradation products, detoxification of algal defense compounds by *Z. galactanivorans* might partly explain the cooperative interaction we showed here with *T. aestuarii*. Further investigations must be pursued to characterize *Z. galactanivorans* interactions with its opportunistic partners and assess whether they rely on public good secretion, cross-feeding interactions and/or even physical mechanisms.

Specific to *Bacteroidetes*, T9SS is involved in biofilm formation, protease virulence factors delivery and secretion of polysaccharidases and cell-surface gliding motility adhesins [98, 99]. Here, we showed that growth with macroalgae strongly induced genes encoding T9SS components, T9SS-translocated proteins and several glycosyl transferases from families GT2 and GT4. Glycosyltransferases with a GT4_CapM-like domain were recently shown to N-glycosylate CTD in *Cytophaga hutchinsonii*, an essential step for the recognition of cargo proteins by T9SS [100]. Hence, our data suggest T9SS might be a key determinant of pioneer behavior for some members of the *Bacteroidetes* phylum, to secrete ECM-targeting CAZymes and/or attach to macroalgal surfaces. Only induced in the presence of algal tissues, this T9SS system might not be triggered by oligo-alginate or oligo-FCSP, but rather by other algal metabolites, such as ROS.

## Conclusion

This study provides the first insights into the metabolic strategies of sharing pioneer bacteria during fresh macroalgae utilization and represents a source of potential genetic determinants for further functional characterization. Altogether, our results raised the relevance to consider the full complexity of whole macroalgae tissues in further degradation studies, as it would take a step forward in the understanding of the algal biomass recycling through the identification of new metabolic pathways or the characterization of bacterial cooperative interactions. Integrative time-series investigations would be particularly helpful to bring a more comprehensive view of the strategies taking place during algal breakdown.

## Supplementary information

Supplementary Material

Supp Table 4

Supp Table 5

Supp Table 7
